# Clinical study on the therapeutic effect of large-area cupping therapy for lumbar disc herniation of cold-damp type (a Traditional Chinese Medicine syndrome)

**DOI:** 10.3389/fmed.2026.1742100

**Published:** 2026-05-15

**Authors:** Yingxia Ding, Qian Li, Xinning Liu, Mingdi Liu, Hong Wang, Keqin Zhao, Kaiquan Xu, Duanliang Tian

**Affiliations:** Department of Tuina, Qingdao Traditional Chinese Medicine Hospital, Qingdao Hiser Hospital Affiliated of Qingdao University, Qingdao, China

**Keywords:** chronic low back pain, large-area cupping therapy, lumbar disc herniation, pain score, Prostaglandin E2, randomized controlled trial, β-endorphin

## Abstract

**Background:**

Cupping therapy is a commonly used Traditional Chinese Medicine (TCM) modality for treating lumbar disc herniation (LDH) and has gained increasing attention in recent years. Large-area cupping therapy is characterized by extensive and deep negative-pressure stimulation, which may exert analgesic effects by improving local circulation and modulating inflammatory mediators. This study aimed to evaluate the clinical efficacy of large-area cupping therapy in patients with cold-damp type LDH and to explore its potential mechanisms of action.

**Methods:**

This prospective, randomized controlled trial enrolled 60 patients diagnosed with LDH of the cold-dampness type. Participants were randomly assigned (1:1) using a random number table to either the observation group (*n* = 30) or the control group (*n* = 30). The observation group received large-area cupping therapy, while the control group underwent conventional infrared therapy. Both groups were treated once every other day for a total of six sessions. Pain was assessed using the Visual Analog Scale (VAS), and TCM symptom scores were recorded. Serum levels of prostaglandin E_2_ (PGE_2_) and β-endorphin (β-EP) were measured before treatment, after the first session, and after the final session.

**Results:**

There were no significant differences between the two groups at baseline in terms of VAS scores, TCM symptom scores, or serum levels of PGE_2_ and β-EP (*P* > 0.05). After treatment, the observation group showed significantly greater improvements in VAS scores, symptom scores, and biochemical markers compared to the control group (*P* < 0.05). Specifically, PGE_2_ levels in the treatment group decreased from 123.45 ± 15.93 pg/mL at baseline to 69.79 ± 16.44 pg/mL after treatment, while β-EP levels increased from 4.38 ± 1.09 ng/mL to 7.74 ± 1.12 ng/mL. The total effective rate was 93.33% in the observation group and 83.33% in the control group, with a between-group difference of 10.00%; however, this difference was not statistically significant (*P* = 0.424). No serious adverse events occurred.

**Conclusion:**

Large-area cupping therapy demonstrates promising short-term clinical efficacy in relieving pain and modulating inflammatory mediators in cold-damp type LDH. While safe and well-tolerated, further studies with long-term follow-up are required to establish the durability of these effects.

## 1 Introduction

Lumbar disc herniation (LDH) is one of the most common degenerative spinal disorders encountered in clinical practice. It is primarily characterized by lower back and leg pain, numbness, and restricted mobility, which, in severe cases, can significantly impair patients’ daily functioning and quality of life. Contemporary research suggests that the pathogenesis of LDH is closely related to intervertebral disc degeneration, local biomechanical imbalance, and the release of inflammatory mediators (1). Among the various clinical subtypes, the cold-damp type is particularly prevalent, typically presenting with symptoms such as cold and aching pain in the lower back, exacerbation upon exposure to cold, limited flexion and extension, and restricted movement. This subtype often manifests with a slow onset, prolonged disease course, and a tendency for recurrence (2).

In recent years, increasing evidence has highlighted the pivotal role of inflammation in the pathogenesis of LDH. When the nucleus pulposus herniates into the epidural space, it can trigger an immune-inflammatory cascade, leading to the release of multiple pro-inflammatory mediators such as tumor necrosis factor-alpha (TNF-α), interleukins, and prostaglandin E_2_ (PGE_2_). These mediators contribute to the activation of nociceptors in peripheral nerve endings, thereby initiating or exacerbating pain symptoms (3). Among them, PGE_2_, a key product of the cyclooxygenase pathway, exerts potent pro-nociceptive effects and is considered a crucial biochemical mediator in the development of low back and radicular pain (4). In contrast, β-endorphin (β-EP), an endogenous opioid peptide primarily released from the pituitary and central nervous system, plays a vital role in modulating analgesia. Changes in β-EP levels are thought to reflect the body’s intrinsic pain regulation capacity (5). Therefore, targeting the modulation of PGE_2_ and β-EP levels may offer a promising strategy for alleviating inflammation and pain in patients with LDH.

Currently, Western medical treatments for LDH primarily include non-steroidal anti-inflammatory drugs (NSAIDs), neuromodulators, physical therapy, and minimally invasive surgical interventions. While these approaches can provide short-term symptom relief, long-term pharmacological use may result in gastrointestinal discomfort, renal impairment, and other adverse effects. Surgical interventions, although effective in certain cases, are associated with significant trauma, high cost, and a considerable risk of postoperative recurrence (6, 7). Consequently, an increasing number of patients are turning to TCM therapies, which are considered safer, more economical, and environmentally friendly, as either complementary or alternative treatments (8).

Cupping therapy, a classic external modality in TCM, is believed to “promote Qi and blood circulation, dispel wind and cold, and relax tendons and meridians,” making it particularly suitable for low back pain caused by cold-damp obstruction syndromes (9). Large-area cupping therapy is an evolved technique based on traditional cupping. It is characterized by a wider treatment area, a greater number of cups, and stronger negative pressure stimulation. These features allow for broader and deeper activation of local meridians and microcirculation within a short period, thereby facilitating fluid metabolism, modulating neurohumoral factors, and enhancing analgesic and anti-inflammatory effects.

Previous studies have demonstrated that cupping therapy may be effective in the management of LDH. For instance, a randomized three-arm trial conducted by Teut et al. involving 110 patients with chronic low back pain reported a significant reduction in visual analog scale (VAS) scores after 4 weeks of pulsed dry cupping compared to controls (*P* < 0.001) (10). A more recent study in 2023 found that mobile cupping alleviated chronic low back pain by improving muscle tension and enhancing local blood flow (11). Furthermore, a randomized controlled clinical trial demonstrated that dry cupping therapy had a positive effect on pain and functional disability in patients with persistent non-specific low back pain, showing superior improvement in pain relief and functional scores compared with the control group (12). Additionally, a systematic review on the “Fire Dragon Cupping” method suggested its clinical value in the treatment of LDH (13).

In parallel with growing clinical use, a substantial body of recent literature has sought to place cupping therapy within an evidence-based biomedical framework. Multiple systematic reviews and meta-analyses have demonstrated that cupping—particularly for musculoskeletal pain and chronic low back pain—produces clinically meaningful reductions in pain intensity compared with usual care, wait-list control, or physical modalities, although heterogeneity in techniques, dosing, and outcome measures remains high (14–17). These syntheses collectively indicate that cupping is not merely a traditional practice but a modality with reproducible analgesic effects across diverse clinical settings, while underscoring the need for better standardization of technique and treatment parameters.

Beyond clinical efficacy, mechanistic studies have increasingly clarified how cupping may exert its effects. Experimental and clinical evidence suggests that negative pressure induces local microvascular responses, enhances tissue perfusion, modulates lymphatic drainage, and alters the local inflammatory milieu. Reviews of wet cupping, in particular, have highlighted reductions in pro-inflammatory mediators and shifts in biochemical profiles related to oxidative stress and immune activation (18, 19). Parallel work in dry cupping has shown changes in inflammatory markers following high-intensity exercise, supporting a generalized anti-inflammatory influence that extends beyond localized tissue effects (20).

Comparative research on dry versus wet cupping further suggests that technique-specific mechanisms may differ: wet cupping may exert stronger effects on systemic inflammatory markers, whereas dry cupping appears to act more prominently through mechanical decompression, fascial mobility, and neuromodulation (21, 22). Dose-related factors—such as negative pressure magnitude, treated surface area, and retention time—also appear to shape both physiological responses and clinical outcomes (15, 23).

Although growing evidence supports the integration of cupping into evidence-informed pain management, key gaps remain. The mechanisms and clinical value of large-area cupping are insufficiently studied, and few investigations have focused on cold-damp type LDH. Moreover, direct clinical evidence linking cupping to changes in PGE_2_ and β-endorphin in LDH patients is still limited. Therefore, the present study employed a randomized controlled design to evaluate the clinical efficacy of large-area cupping therapy in patients with cold-damp type LDH. By assessing changes in pain scores, functional symptoms, and serum levels of PGE_2_ and β-EP, we aimed to preliminarily explore the underlying mechanisms and provide evidence-based support for its clinical application.

## 2 Materials and methods

### 2.1 Study design and participants

This was a single-center, prospective, randomized controlled trial conducted between January 2023 and June 2025 at the Department of Tuina, Qingdao Hospital of Traditional Chinese Medicine. A total of 60 patients diagnosed with LDH associated with chronic low back pain and classified as cold-damp type according to TCM criteria were enrolled. All participants provided written informed consent prior to enrollment. The study protocol was reviewed and approved by the Institutional Ethics Committee of the hospital (Approval Number: 2022HC05LS023). Eligible participants were randomly assigned to either the treatment group or the control group using a random number table, with 30 patients in each group. The random sequence was generated by an independent statistician who was not involved in the recruitment or treatment of participants. To ensure allocation concealment, the assignments were placed in sequentially numbered, opaque, sealed envelopes. These envelopes were opened by a designated research assistant only after the participant had completed the baseline assessment and met all inclusion criteria.

### 2.2 Sample size estimation

As an exploratory prospective randomized controlled study, the sample size was preliminarily estimated based on prior randomized controlled trials and literature-reported differences in VAS scores after treatment (10). Assuming an effect size (Cohen’s *d*) of approximately 0.8 between groups, with a significance level (α) of 0.05 and power (1–β) of 0.8, a minimum of 26 participants per group were required as calculated using G*Power 3.1 software. Considering a dropout rate of approximately 10%, the final sample size was set at 30 participants per group, totaling 60 subjects.

### 2.3 Diagnostic criteria

#### 2.3.1 Western medicine diagnostic criteria

The diagnosis of LDH in this study was established based on the evidence-based clinical guidelines titled *Diagnosis and Treatment of Lumbar Disc Herniation with Radiculopathy* issued by the North American Spine Society (NASS) (24), in combination with clinical manifestations, physical examination, and imaging findings. The diagnostic criteria were as follows:

*Chronic low back pain history:* Persistent or recurrent low back pain lasting ≥ 12 weeks;
*Typical clinical symptoms:* Lumbar pain radiating to the buttocks or lower extremities, consistent with sciatic nerve distribution;
*Positive physical signs:* Restricted lumbar mobility, paraspinal tenderness, and positive straight-leg raise (SLR) test or enhanced SLR test, indicating nerve root compression;
*Imaging support:* Radiographic evidence (X-ray, CT, or MRI) of disc degeneration, narrowed intervertebral space, disc herniation, or nerve root compression adjacent to the affected segment.

#### 2.3.2 TCM Syndrome differentiation criteria

The diagnostic criteria for cold-damp type low back pain were formulated with reference to the *Standards for Diagnosis and Efficacy of TCM Diseases and Syndromes* (25). Key features include pronounced cold pain in the lower back, exacerbation upon exposure to cold, stiffness during turning movements, poor relief from bed rest, worsening symptoms on rainy or damp days, pale tongue with white greasy coating, and a deep-tight or soft pulse. Syndrome differentiation was independently performed by two senior TCM physicians, each with over 15 years of clinical experience. To ensure diagnostic consistency, an inter-rater reliability assessment was conducted prior to the study, yielding a Kappa coefficient (κ) of 0.88. In cases of disagreement, a third senior physician was consulted for adjudication; in this study, four cases (approximately 6% of the total screened participants) required such adjudication to reach a final consensus. Syndrome differentiation was independently performed by two senior TCM physicians. In cases of disagreement, a third physician was consulted for adjudication. In this study, four cases (approximately 6% of the total screened participants) required adjudication by a third senior physician to reach a final consensus.

### 2.4 Inclusion and exclusion criteria

Inclusion criteria:

(1) Meeting both the Western medicine diagnostic criteria for LDH and the TCM cold-damp syndrome differentiation;
(2) Aged between 21 and 76 years, regardless of sex;
(3) Chronic low back pain with a duration of ≥ 12 weeks, supported by imaging findings;
(4) No other treatments (e.g., medications, physical therapy, external TCM interventions) received within the past month;
(5) Voluntary participation with signed informed consent.

Exclusion criteria:

(1) Specific causes of low back pain such as spinal tumors, tuberculosis, spinal stenosis, or fractures;
(2) History of lumbar spine surgery;
(3) Presence of severe cardiovascular, cerebrovascular, hepatic, renal dysfunction, or psychiatric disorders;
(4) Pregnant, breastfeeding, or menstruating women;
(5) Contraindications to cupping or infrared therapy.

Dropout criteria:

(1) Poor treatment compliance;
(2) Withdrawal from the study for any reason;
(3) Loss to follow-up or incomplete outcome assessment.

### 2.5 Intervention methods

#### 2.5.1 Observation group (large-area cupping therapy)

**FIGURE 1:**
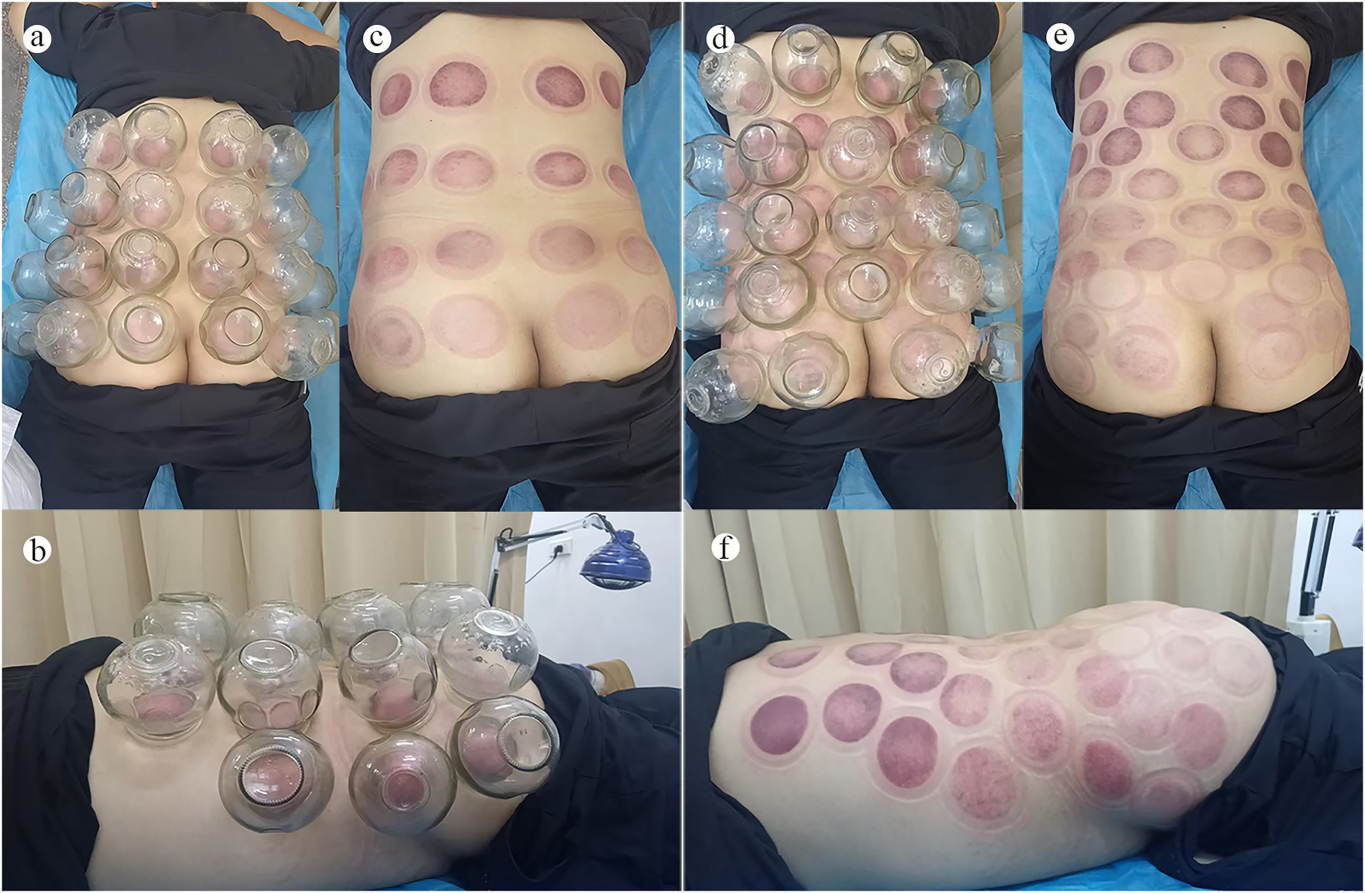
Application and effects of large-area cupping therapy in a patient with lumbar disc herniation. **(a)** First cupping session: anterior view of the cup distribution on the patient’s lower back. **(b)** First session: lateral view of the cup placement. **(c)** After the first cupping session: marks on the treated area (posterior view). **(d)** Second cupping session: cup distribution on the patient’s back (anterior view). **(e)** After the second cupping session: posterior view of the cupping marks. **(f)** After the second session: lateral view of the treated area with visible circular marks.

Patients were positioned prone to expose the lumbosacral and gluteal regions. Prior to treatment, routine skin disinfection and preparation of the cupping apparatus were performed. Cup size was selected by the treating clinician according to the participant’s lumbar body build and soft tissue thickness, and the choice was recorded during treatment. Based on body type, No. 4 glass cups were used for slender individuals and No. 5 cups for overweight individuals. To ensure treatment standardization, the negative pressure was maintained between −0.04 and −0.06 MPa (approximately 300–450 mmHg), which was monitored and verified using digital pressure gauges integrated into the vacuum system. During treatment, a sterile cotton ball soaked in 95% ethanol was clamped with forceps, ignited by an assistant, and briefly (2–5 s) inserted into the cup to heat the air inside. The cotton ball was then removed immediately, and the heated cup was quickly applied to the skin to create negative pressure suction. The cupping sites included both sides of the paraspinal region from the first lumbar vertebra to the sacrum, the sacrococcygeal area, and the bilateral gluteal region. To ensure full coverage of the treatment area, cupping was performed in two rounds. After the primary sites were cupped in the first round, any remaining uncovered regions were treated in a second round (Figure 1). Each session involved static cupping for 10 min, performed once every other day. A total of 6 sessions constituted one treatment course.

All treatments were performed by three licensed TCM practitioners, each with over 5 years of clinical experience in spinal disorders. Prior to the study, all practitioners completed a 40-h intensive training program on the standardized protocol. Inter-practitioner reliability was formally assessed, yielding a Kappa coefficient (κ) of 0.85, indicating high consistency in treatment delivery.

#### 2.5.2 Control group (infrared therapy)

Participants were placed in the same prone position with the lumbosacral area exposed. An infrared therapy device was positioned 30–50 cm above the skin. The distance and intensity were adjusted to ensure a warm, comfortable sensation without burning. Each treatment lasted 30 min, administered once every other day, for a total of 6 sessions per course.

### 2.6 Outcome measures

#### 2.6.1 Pain intensity (VAS score)

Pain intensity was assessed using the VAS, which ranges from 0 to 10, with higher scores indicating more severe pain. Evaluations were conducted at three time points: before treatment, after the first session, and after the final session.

#### 2.6.2 TCM Syndrome score

Based on the “Guiding Principles for Clinical Evaluation of New Chinese Medicines” (26), syndrome scoring was performed from four aspects: severity of low back pain, range of motion limitation, accompanying symptoms (e.g., stiffness, numbness), and aggravating factors. Each domain was scored on a four-point ordinal scale from 0 to 3, where 0 indicates no symptom and 3 indicates the most severe presentation; the total score therefore ranges from 0 to 12, with higher scores indicating more severe symptoms. Because aggravation by cold or damp exposure is part of the syndrome-defining profile for cold-damp LDH, a score of 2 for aggravating factors at baseline was plausible across all enrolled patients, while a score of 1 after treatment indicates mild residual symptoms and can also result in a standard deviation of 0 when all participants fall in the same category.

#### 2.6.3 Laboratory parameters

Fasting venous blood samples (3 mL) were collected before treatment, after the first session, and after the final session. Serum was separated and levels of prostaglandin E2 (PGE_2_) and β-endorphin (β-EP) were measured using enzyme-linked immunosorbent assay (ELISA). ELISA optical-density output was converted to concentrations according to the standard curve and then normalized in a unit-consistent spreadsheet (ng/mL for β-EP) before statistical analysis. To improve consistency, all blood samples were collected in the morning under fasting conditions at the same predefined time window. Post-treatment blood collection was performed 30 min after the end of the corresponding treatment session.

All clinical outcomes were assessed by two independent researchers who were intended to remain blinded to the group assignments of the participants. These assessors were not present during the treatment sessions and did not have access to the randomization records; however, because visible cupping marks could remain on the skin after treatment, complete assessor blinding during physical examination could not be guaranteed.

#### 2.6.4 Safety assessment

Adverse events (AEs) were systematically monitored and recorded at each treatment session. In this study, an AE was defined as any untoward and unintended sign, symptom, or disease temporally associated with the treatment, such as skin blisters, thermal burns, persistent localized pain, or systemic discomfort. Notably, the transient skin ecchymosis or petechiae (cupping marks) resulting from negative pressure was classified as a characteristic therapeutic response rather than an adverse event, provided there was no skin breakdown or infection.

### 2.7 Criteria for clinical efficacy evaluation

Clinical efficacy was evaluated based on the *Standards for Diagnosis and Therapeutic Effect of Diseases and Syndromes in TCM* (25). The criteria were defined as follows:

*Cured****:*** Complete resolution of low back and leg pain; VAS reduction ≥ 95%; normal mobility; straight leg raise (SLR) ≥ 80°.
*Markedly effective****:*** Significant symptom improvement; VAS reduction ≥ 70%; mild limitation of movement; SLR 70°–79°.
*Effective:* Partial relief; VAS reduction ≥ 30%; SLR < 70°.
*Ineffective:* VAS reduction < 30% or no clinical improvement.
Total effective rate = (Number of cured + markedly effective + effective cases) ÷ total number of cases × 100%.

### 2.8 Statistical analysis

All data were analyzed using SPSS version 26.0 (IBM Corp., Armonk, NY, United States). The Shapiro–Wilk test was used to assess normality. Measurement data with a normal distribution were expressed as mean ± standard deviation. For longitudinal data measured at three time points (before treatment, after the first session, and after the final session), a repeated-measures analysis of variance (ANOVA) was employed. The model incorporated group as the between-subjects factor and time as the within-subjects factor. Sphericity was assessed using Mauchly’s test; if the assumption was violated (*P* < 0.05), the Greenhouse-Geisser correction was applied to adjust the degrees of freedom. To identify specific differences, *post-hoc* pairwise comparisons were conducted with Bonferroni correction to maintain the family-wise Type I error rate at 0.05. Significant group-by-time interactions were further explored to determine whether the rate of clinical improvement differed between the two interventions. Baseline characteristics were compared using independent samples *t*-tests or chi-square tests. Categorical data were presented as frequencies and percentages. Data analysis was performed by a third-party statistician who remained blinded to the treatment allocation until the completion of the analysis. A two-sided *P* < 0.05 was considered statistically significant.

## 3 Results

### 3.1 Baseline characteristics

A total of 60 participants were enrolled and randomly assigned to either the treatment group (*n* = 30) or the control group (*n* = 30). As shown in Table 1, there were no significant differences between the two groups in demographic data, including age, sex, and BMI (*P* > 0.05). Furthermore, the groups were well-balanced regarding clinical characteristics, such as disease duration, herniation level, neurological symptoms, and baseline VAS scores (*P* > 0.05). These results confirm the comparability of the two groups prior to the intervention (Table 1).

**TABLE 1:** Baseline characteristics of the two groups.

Characteristics	Treatment Group (*n* = 30)	Control group (*n* = 30)	*t*/χ ^2^	*P*-value
Age, years	45.47 ± 5.67	36.70 ± 11.02	1.841	0.071
Sex (Male/Female)	17/13	12/18	1.684	0.194
BMI, kg/m^2^	24.12 ± 2.34	23.85 ± 2.51	0.431	0.668
Disease duration, months	14.23 ± 5.12	13.87 ± 4.95	0.277	0.783
Herniation level, n (%)			0.516	0.773
L3/4	4 (13.33%)	3 (10.00%)		
L4/5	18 (60.00%)	19 (63.33%)		
L5/S1	8 (26.67%)	8 (26.67%)		
VAS score	7.10 ± 1.16	6.97 ± 1.10	0.445	0.658
Neurological symptoms, n (%)			0.144	0.705
Numbness	22 (73.33%)	21 (70.00%)		
Weakness	8 (26.67%)	9 (30.00%)		

BMI, Body Mass Index; VAS, Visual Analogue Scale. Values are expressed as mean ± standard deviation or number (percentage). Continuous variables were analyzed using the independent-samples t test, and categorical variables were analyzed using the χ^2^ test. No significant differences were observed between the two groups at baseline (*P* > 0.05).

### 3.2 Comparison of VAS scores between the two groups

Repeated-measures ANOVA was conducted to analyze the changes in VAS scores across three time points. The results revealed a significant main effect of time (*F* = 245.62, *P* < 0.001), indicating that pain intensity decreased in both groups over the course of the study. A significant main effect of group was also observed (*F* = 12.45, *P* = 0.001), with the treatment group exhibiting lower overall pain scores than the control group. Importantly, a significant group-by-time interaction effect was identified (*F* = 8.92, *P* = 0.004), demonstrating that the rate of pain reduction was significantly greater in the large-area cupping group compared to the control group (Table 2). Post-hoc pairwise comparisons with Bonferroni adjustment showed no significant between-group difference in VAS scores at baseline (*P* > 0.05). After treatment, VAS scores were significantly reduced compared with baseline at both post-treatment time points in both groups (*P* < 0.05). Moreover, the treatment group demonstrated significantly lower VAS scores than the control group after both the first and final treatments (*P* < 0.05), supporting the superior analgesic effect of large-area cupping therapy for LDH-related pain (Table 3).

**TABLE 2:** Repeated-measures ANOVA results for VAS.

Effect	F	*P* value	$\eta_{\text{p}}^{2}$
Group	12.45	0.001	0.17
Time	245.62	< 0.001	0.81
Group × Time	8.92	0.004	0.13

$\eta_{\text{p}}^{2}$ = partial eta squared (effect size).

**TABLE 3:** Comparison of VAS scores between the two groups.

Group	n	Before treatment	After first treatment	After final treatment
Treatment	30	7.10 ± 1.16	4.03 ± 1.10^a,b^	3.10 ± 1.20^a,b^
Control	30	6.97 ± 1.10	5.07 ± 1.14^a^	4.05 ± 1.27^a^

VAS, Visual Analog Scale. Values are presented as mean ± standard deviation.

^a^*P* < 0.05 vs. before treatment (within-group comparison).

^b^*P* < 0.05 vs. the control group (between-group comparison). At the final treatment, the mean difference between groups was −0.95 (95% CI: −1.59 to −0.31), with a Cohen’s d of 0.77. Although this difference was statistically significant, it remained below commonly cited minimum clinically important difference thresholds of 1.5–2.0 points for pain and should therefore be interpreted cautiously with respect to clinical meaningfulness.

### 3.3 Comparison of TCM symptom scores

Separate repeated-measures ANOVAs were conducted for each of the four TCM symptom dimensions (low back pain, limited mobility, accompanying symptoms, and aggravating factors). The analysis revealed significant main effects of time (all *P* < 0.001) and group (all *P* ≤ 0.004) across all symptom domains, indicating overall symptom improvement over time and lower scores in the treatment group compared with the control group. Importantly, significant group × time interaction effects were observed for low back pain (*F* = 9.45, *P* = 0.000), limited mobility (*F* = 8.12, *P* = 0.001), accompanying symptoms (*F* = 7.38, *P* = 0.001), and aggravating factors (*F* = 6.15, *P* = 0.003). These findings indicate that the trajectory of symptom improvement was significantly more favorable in the large-area cupping group than in the infrared group (Table 4). *Post-hoc* pairwise comparisons with Bonferroni adjustment showed no significant between-group differences at baseline for any TCM symptom score (*P* > 0.05). After treatment, all four symptom dimensions decreased significantly from baseline at both post-treatment time points in both groups (*P* < 0.05). Furthermore, at both the first and final post-treatment sessions, the treatment group consistently exhibited significantly lower scores than the control group across all four symptom domains (*P* < 0.05), supporting the superior clinical effect of large-area cupping (Table 5).

**TABLE 4:** Repeated-measures ANOVA results for TCM syndrome scores.

Outcome	Effect	*F*	*P*-value	$\eta_{\text{p}}^{2}$
Low back pain	Group	12.14	0.001	0.17
	Time	215.36	< 0.001	0.79
	Group × Time	9.45	0.000	0.14
Limited mobility	Group	10.82	0.002	0.16
	Time	198.67	< 0.001	0.77
	Group × Time	8.12	0.001	0.12
Accompanying symptoms	Group	9.56	0.003	0.14
	Time	165.24	< 0.001	0.74
	Group × Time	7.38	0.001	0.11
Aggravating factors	Group	8.94	0.004	0.13
	Time	142.85	< 0.001	0.71
	Group × Time	6.15	0.003	0.10

$\eta_{\text{p}}^{2}$ = partial eta squared (effect size).

**TABLE 5:** Comparison of TCM syndrome scores between the two groups.

Group	Time	Low back pain	Limited mobility	Accompanying symptoms	Aggravating factors
Treatment	Before treatment	2.87 ± 0.46	2.50 ± 0.51	2.30 ± 0.47	2.00 ± 0.00
	After first treatment	2.03 ± 0.32^a,b^	1.73 ± 0.45^a,b^	1.47 ± 0.51^a,b^	1.17 ± 0.38^a,b^
	After final treatment	1.20 ± 0.41^a,b^	1.00 ± 0.00^a,b^	0.70 ± 0.47^a,b^	0.35 ± 0.49^a,b^
Control	Before treatment	2.70 ± 0.47	2.30 ± 0.47	2.03 ± 0.49	1.90 ± 0.31
	After first treatment	1.97 ± 0.18^a^	1.77 ± 0.43^a^	1.40 ± 0.50^a^	1.10 ± 0.31^a^
	After final treatment	1.63 ± 0.50^a^	1.32 ± 0.48^a^	1.10 ± 0.32^a^	0.95 ± 0.23^a^

TCM, Traditional Chinese Medicine. Values are presented as mean ± standard deviation. Each domain was scored on an ordinal scale from 0 to 3; therefore, an SD of 0.00 can occur when all participants fall in the same category. In the present dataset, all treatment-group participants scored 2 for aggravating factors at baseline and 1 for limited mobility at the final assessment.

^a^*P* < 0.05 vs. before treatment (within-group comparison).

^b^*P* < 0.05 vs. the control group (between-group comparison). At the final session, the between-group mean difference for the total TCM score was −1.48 (95% CI: −1.92 to −1.04).

### 3.4 Changes in serum prostaglandin E2 and β-endorphin levels

Separate repeated-measures ANOVAs were conducted for serum PGE_2_ and β-EP levels. For PGE_2_, significant main effects were identified for group (*F* = 9.82, *P* = 0.003) and time (*F* = 156.34, *P* < 0.001), along with a significant group-by-time interaction (*F* = 6.45, *P* = 0.003). For β-EP, the analysis revealed a significant increase over time (*F* = 112.58, *P* < 0.001) and a significant group-by-time interaction (*F* = 5.92, *P* = 0.006), indicating that the longitudinal pattern of change differed between groups (Table 6). Post-hoc comparisons showed that before treatment, no significant differences existed between groups (*P* > 0.05). Following treatment, PGE_2_ levels decreased while β-EP levels increased significantly within both groups (*P* < 0.05). The treatment group showed a significantly greater reduction in PGE_2_ at both post-treatment time points compared with the control group (Table 7). For β-EP, however, the between-group mean difference at the final assessment was 0.11 ng/mL (95% CI: −0.44 to 0.66), indicating that the final pairwise difference did not reach statistical significance despite the significant interaction over time. These findings suggest that large-area cupping therapy effectively reduces inflammatory activity, while its effect on β-EP is better interpreted as a differential temporal trend rather than a clearly significant end-point difference.

**TABLE 6:** Repeated-measures ANOVA results for serum biomarkers.

Outcome	Effect	*F*	*P*-value	$\eta_{\text{p}}^{2}$
PGE_2_	Group	9.82	0.003	0.14
	Time	156.34	< 0.001	0.73
	Group × Time	6.45	0.003	0.10
β-EP	Group	8.12	0.005	0.12
	Time	112.58	< 0.001	0.66
	Group × Time	5.92	0.006	0.09

$\eta_{\text{p}}^{2}$ = partial eta squared (effect size).

**TABLE 7:** Comparison of serum PGE_2_ and β-endorphin levels between the two groups before and after treatment.

Group	Time	PGE_2_(pg/mL)	β−EP (ng/mL)
Treatment	Before treatment	123.45 ± 15.93	4.38 ± 1.09
	After first treatment	101.51 ± 13.91^a,b^	6.09 ± 1.12^a^
	After final treatment	69.79 ± 16.44^a,b^	7.74 ± 1.12^a^
Control	Before treatment	123.63 ± 16.07	4.99 ± 0.97
	After first treatment	103.84 ± 18.54^a^	6.50 ± 1.06^a^
	After final treatment	83.67 ± 15.44^a^	7.63 ± 1.08^a^

PGE_2_, prostaglandin E2; β-EP, β-endorphin. Values are presented as mean ± standard deviation.

^a^*P* < 0.05 vs. before treatment (within-group comparison).

^b^*P* < 0.05 vs. the control group (between-group comparison). At the final assessment, the mean difference (95% CI) between groups was −13.88 (-22.14 to −5.62) for PGE_2_ and 0.11 (-0.44 to 0.66) for β-EP.

### 3.5 Comparison of clinical efficacy

According to the intention-to-treat (ITT) analysis, the total effective rate was 93.33% (28/30) in the treatment group and 83.33% (25/30) in the control group. Although the total effective rate was numeric ally higher in the treatment group, the between-group difference was not statistically significant (*P* = 0.424) (Table 8).

**TABLE 8:** Comparison of clinical efficacy between the two groups.

Group	n	Recovery (n)	Marked effect (n)	Improvement (n)	No effect (n)	Total effective rate (%)	95% CI (%)
Treatment	30	21	4	3	2	93.33	77.93, 99.18
Control	30	18	3	4	5	83.33	65.28, 94.36

Total effective rate = [(Recovery + Marked Effect + Improvement)/Total cases] × 100%. The between-group difference was not statistically significant (Pearson χ^2^ = 1.46, *P* = 0.227; Fisher’s exact test, *P* = 0.424).

### 3.6 Safety and compliance evaluation

A total of 62 participants were initially enrolled, with two dropouts: one in the treatment group due to an unrelated medical condition and one in the control group lost to follow-up. Ultimately, 30 patients per group completed the study. No serious adverse events (AEs), such as skin burns or blisters, occurred. The localized skin ecchymosis observed in the treatment group (Figure 1) was classified as an expected therapeutic response to negative pressure rather than an AE, and it resolved spontaneously within 3–7 days. Two patients reported transient soreness that subsided within 24 h. These results indicate that large-area cupping therapy is safe and well-tolerated.

## 4 Discussion

LDH is a common degenerative spinal disorder, with chronic low back pain significantly impairing patients’ quality of life and functional status. In TCM, the cold-damp type is a frequent syndrome of LDH, typically characterized by symptoms such as cold pain in the lower back, limited mobility, and aggravation with cold exposure. The underlying pathogenesis is believed to involve obstruction of meridians by cold-damp pathogens, resulting in impaired circulation of qi and blood. Therefore, treatment should focus on warming the meridians, dispelling cold and dampness, and alleviating pain.

This prospective comparative effectiveness study evaluated the clinical outcomes of large-area cupping therapy relative to infrared therapy in patients with cold-damp type LDH. The results demonstrated that, compared to conventional infrared therapy, large-area cupping significantly improved pain relief, TCM syndrome scores, and inflammatory-marker profiles. Furthermore, although it showed a higher total effective rate with good safety and tolerance, the between-group difference did not reach statistical significance. These findings suggest that this therapy may be a promising treatment option for this patient population.

Large-area cupping therapy applies negative pressure stimulation to the lumbosacral and lower limb regions, generating mechanical traction on the skin, musculature, and fascial tissues. This stimulation improves local blood circulation and lymphatic drainage, enhances tissue oxygenation and metabolic activity, and facilitates the clearance of inflammatory mediators. These physiological changes contribute to the alleviation of soft tissue tension and adhesion (27). Compared with traditional localized cupping, the large-area technique provides broader stimulation coverage and stronger cumulative mechanical input, which may amplify both local and systemic physiological responses.

Recent research has advanced understanding of cupping therapy beyond traditional explanations. A comprehensive review by Dergaa et al. highlighted that wet cupping can modulate inflammatory pathways, enhance microcirculation, and influence pain-related biochemical mediators, mechanisms that may also apply to dry and large-area cupping techniques (18). In addition, sports medicine studies have shown that both wet and dry cupping improve perceived wellness, reduce perceived exertion, and modulate inflammatory responses. Dergaa et al. reported enhanced endurance performance and subjective wellness in recreational runners, while Irandoust et al. demonstrated altered inflammatory and cardiovascular responses following dry cupping in athletes (20, 28). Collectively, these findings suggest that cupping therapy exerts not only local tissue effects but also systemic biochemical and neurophysiological modulation, which may share common central and peripheral mechanisms with pain relief in chronic low back pain.

In the present study, a significant reduction in serum PGE_2_ levels was observed following cupping treatment, suggesting a potential anti-inflammatory effect. This finding is consistent with emerging experimental evidence indicating that cupping therapy can modulate inflammatory markers under both pathological and stress-induced conditions. For example, Irandoust et al. demonstrated that dry cupping significantly altered inflammatory and cardiovascular responses in athletes exposed to high-intensity exercise, supporting the hypothesis that cupping exerts a generalized anti-inflammatory influence across different physiological contexts (28). Although their study focused on exercise-induced inflammation, while the present study examined chronic inflammatory processes associated with LDH, both contexts involve inflammatory mediator activation, indicating potential shared mechanisms.

β-EP, an endogenous opioid peptide primarily released from the pituitary gland and central nervous system, plays a key role in pain modulation. In this study, serum β-EP levels increased over time in both groups, and the repeated-measures analysis suggested a different temporal pattern of change between interventions. However, the final between-group difference in β-EP was small and not statistically significant. Therefore, the β-EP findings should be interpreted cautiously: they support a possible contribution of endogenous opioid regulation to the observed analgesic response, but they do not by themselves establish a clearly superior end-point effect of large-area cupping on this biomarker. This more conservative interpretation is consistent with the corrected dataset and with the observed reduction in the pro-inflammatory mediator PGE_2_ (29).

Beyond biochemical modulation, recent sports medicine studies have provided important insights into the perceptual and neurophysiological effects of cupping therapy. Dergaa et al. reported that both wet and dry cupping significantly improved perceived wellness, reduced rating of perceived exertion, and enhanced endurance performance in recreational athletes (20). Although perceived exertion and pain are distinct constructs, both involve central perceptual processing within overlapping neural networks (30). The observed reductions in VAS pain scores and TCM symptom scores in our study may therefore reflect not only peripheral anti-inflammatory effects but also central modulation of pain perception and symptom awareness.

Previous studies have also provided supportive evidence for the analgesic mechanisms of cupping therapy. Zhang et al. conducted a systematic review including 11 clinical trials with a total of 921 patients with chronic low back pain. The findings demonstrated that cupping therapy significantly reduced pain symptoms within 2–8 weeks, with a standardized mean difference (SMD) of 1.09 (*P* < 0.01) and moderate to high quality of evidence (23). In addition, Harper et al. reported in a case series that a combination of static and dynamic dry cupping improved fascial gliding, reduced muscle tension, and enhanced mobility, thus supporting the analgesic effect of cupping from a biomechanical perspective of soft tissue modulation (31). In the present trial, the final between-group VAS difference was 0.95 points. Although statistically significant, this value remains below commonly cited MCID thresholds of 1.5–2.0 points for pain and should therefore be interpreted as suggesting modest short-term clinical superiority rather than a definitively large clinically meaningful advantage. The magnitude of pain reduction observed in the present study is nevertheless broadly comparable to effect sizes reported in previous meta-analyses, which may be partially attributable to syndrome-specific patient selection and the use of a large-area cupping technique.

Furthermore, in TCM, the core principle in treating cold-damp type low back pain is “dispersing cold and eliminating dampness, warming the meridians, and unblocking collaterals.” The thermal stimulation and deep negative pressure traction generated by large-area cupping are consistent with the TCM theories of “warming for cold” and “unblocking to relieve pain.” This reflects a potential integration between TCM external therapies and modern pathophysiological mechanisms of pain. Recent literature emphasizes the importance of bridging traditional theoretical frameworks with contemporary biomedical evidence, supporting the rational integration of TCM practices into evidence-based pain management strategies (32).

Notably, large-area cupping involves broader application areas and higher stimulation intensity compared with traditional cupping, enabling not only local symptom relief but also systemic modulation by stimulating meridians and promoting qi and blood circulation. This makes it particularly suitable for patients with chronic, recurrent cold-damp low back pain.

However, this study has several limitations. First, the sample size is relatively small. Although post-hoc power analysis confirmed a statistical power > 0.80 for the primary outcome (VAS), the study may be underpowered for certain secondary or categorical measures, potentially increasing the risk of Type II errors. Consequently, these findings should be considered preliminary. Nonetheless, the observed effect sizes ( = 0.09–0.17) suggest that larger confirmatory trials are warranted to further validate the clinical utility of this therapy. Second, a numerical age imbalance existed between groups (mean difference: 8.77 years; 95% CI: 4.06–13.48; *P* = 0.071). Although statistically non-significant, this gap is clinically relevant as age influences disc degeneration and recovery rates. To mitigate potential confounding, we performed a sensitivity analysis using analysis of covariance (ANCOVA) adjusting for age. The results confirmed that the therapeutic superiority of large-area cupping remained significant (*P* < 0.05), indicating that the clinical outcomes were independent of the age distribution. Third, although outcome assessors were intended to be blinded, assessor blinding during physical examination was likely compromised by the visible cupping marks shown in Figure 1. This is a particularly important limitation for subjective outcomes such as VAS and TCM symptom scores, as patient expectations and assessor awareness may have introduced performance and assessment bias. Fourth, the control group received infrared therapy, which differs from large-area cupping in terms of treatment duration, stimulation intensity, and sensory experience. This imbalance may influence patient expectations and introduce potential placebo effects, which could partially affect the perceived treatment outcomes. Although infrared therapy was selected as a standardized active control to provide comparable thermal effects for the cold-damp syndrome, it does not fully account for the non-specific effects of the cupping procedure. Fifth, the treatment efficacy was evaluated only immediately following the 2-week intervention. Given the chronic and recurrent nature of LDH, the lack of long-term follow-up limits our understanding of the therapy’s sustained effects and impact on recurrence rates. Consequently, our findings primarily support the short-term clinical utility of large-area cupping. Sixth, the mechanistic exploration was based on indirect biochemical markers; further studies integrating imaging, electromyography, or molecular indicators are warranted. Additionally, while recent studies have highlighted the benefits of wet cupping on inflammatory and perceptual outcomes, direct comparisons between large-area dry cupping, traditional dry cupping, and wet cupping for different TCM syndrome types remain lacking. Future trials should incorporate sham-controlled or dosage-matched designs to clarify optimal cupping strategies and isolate technique-specific mechanisms.

## 5 Conclusion

Large-area cupping therapy, as a TCM external treatment that integrates traditional principles with modern mechanisms, demonstrates promising clinical efficacy in the management of cold-damp type lumbar disc herniation. Its therapeutic effects may be attributed to its anti-inflammatory and analgesic. The present study provides preliminary evidence for future clinical application and research. Future high-quality studies with multicenter designs, larger sample sizes, more rigorous blinding procedures, sham-controlled comparisons, and long-term follow-up are needed to further validate its efficacy and elucidate the underlying mechanisms.

## Data Availability

The raw data supporting the conclusions of this article will be made available by the authors, without undue reservation.
